# Use of polyurea from urea for coating of urea granules

**DOI:** 10.1186/s40064-016-2120-x

**Published:** 2016-04-14

**Authors:** Panfang Lu, Yanfei Zhang, Cong Jia, Yufeng Li, Zhiquan Mao

**Affiliations:** College of Chemistry and Material Science, Shandong Agricultural University, Tai’an, 271018 Shandong People’s Republic of China; College of Horticulture Science and Engineering, Shandong Agricultural University, Tai’an, 271018 Shandong People’s Republic of China

**Keywords:** Polyurea-coated urea, Urea, Coating, Controlled release fertilizers

## Abstract

A new type of controlled release fertilizers coated with polyurea was prepared. The granulated urea was firstly changed into a liquid urea by heating as the coating liquid. By spraying uniformly the urea was coated with the polyurea synthesized by the reaction of isocyanates with a liquid urea. The effects of different modifiers on N release characteristics of polyurea-coated urea (PCU) were studied. The morphology and chemical structure of PCU coating materials was investigated by SEM and FTIR. We studied the nitrogen release characteristics of the PCU applied in both water and soil, and the biodegradability of PCU coating after buried in soil. The results showed that PCU reduced nitrogen release rate and exhibited excellent controlled release property. The PCU coating materials could biodegrade in soil. This indicated that the low cost PCU products from urea are expected to use in agricultural and horticultural applications.

## Background

Fertilizers are one of the most important products in agriculture. Crop yields and growth mainly depend on the use of fertilizers. But with the increase of the usage amount and time of fertilizers, environmental issue became more conspicuous. It was experimentally confirmed that the application of slow-release or controlled-release fertilizers (CRF), by coating urea granules with materials that exhibit excellent water resisting property, was an effective solution to reduce fertilizer losses and to minimize environmental pollution.

The slow-release or controlled-release fertilizers have been developed over the past decades, which focused on exploring polymer-coated fertilizers. For example, Zhang et al. reported that coating fertilizers nutrients with polyethylene or epoxy resin tended to reduce the nutrient release rate (Lu et al. 2013; Yang et al. 2012). Tomaszewska et al. found that polysulfone coated fertilizers had excellent controlled release property (Tomaszewska and Jarosiewicz 2006; Tomaszewska et al. 2002). However, polymer-coated fertilizers have a disadvantage that large amounts of non-degradable coating materials are left in soil when the nutrients are exhausted. So, degradable polymers coating materials, including grapheme oxide (Zhang et al. 2014), starch (Zhong et al. 2013), polyhydroxybutyrate (Costa et al. 2013), chitosan (Hussain et al. 2012) were widely studied. Although these degradable polymers coated fertilizers may reduce environmental pollution, these nutrient release longevity of natural polymer coating materials as CRFs is short, which will not meet nitrogen supply requirement for field crops. Moreover, these natural polymer coating materials were very expensive, which increased the costs of production. So, the development of low-cost, biodegradable polymer materials with excellent controlled release properties for CRF coating materials is the best solution.

Polyurea is a synthetic polymer from isocyanate and amine compound. It has high mechanical strength, excellent film-forming property, high electric energy density and water resistance property, which was utilized for various fields, including composited materials, aerogel, film, coating, microcapsules, dielectric materials, and steel plates (Holzworth et al. 2013; Hong and Park 1999; Komurlu and Kesimal 2014; Li et al. 2008; Nikishina et al. 1989; Wu et al. 2014). However, urea is rarely used as compound in the preparation of polyurea. Urea is a small molecular amine compound with two amino groups, and it also reacts with isocyanate to synthesize polyurea resin (Zhang and Lu 2014). However, urea was often utilized directly as fertilizer of plant, which has not been used for the preparation of polyurea according to the literature. Especially, little research has been done to explore urea-based polyurea coated fertilizers for agricultural applications.

Based on this background and our previous studies on polymers (Yang et al. 2013; Zhang et al. 2014), a series of novel polyurea-coated urea (PCU) fertilizers was prepared using polyurea synthesized by the reaction of isocyanates with a liquid urea as the main coating material. The morphology and chemical structure of PCU coating materials was investigated by SEM and FTIR. We studied the nitrogen release characteristics of the PCU applied in both water and soil, and the biodegradability of PCU coating after buried in soil. The polyurea derived from urea, which also provided N nutrients after the degradation of the coating materials in soil. We believe this new method will have great potential for developing CRF that provide plants with nutrients and ensure soil quality and crop productivity.

## Methods

### Materials

Commercial urea granules with a particle size in the range of 2–5 mm were used in the experiment, which were produced by Lanhua Coal Mining Group Co., Ltd. (Shanxi, China). The isocyanates (polymethylene polyphenyl isocyanate (PAPI)) with 30.03 wt% NCO group were obtained from Yantai Wanhua Co. Ltd (Shandong, China).All reagents used were CP or AR grade and easily obtained from commercial sources.

### Preparation of PCUs

The granulated urea was firstly heated and changed into a liquid urea (LU). Then LU, PAPI and (or) modifier were mixed uniformly to obtain the coating liquid. Urea particles were heated to 60–90 °C in a rotary drum. Afterwards the above coating liquid was evenly sprayed on the surface of urea particles and cured in a few minutes to synthesize polyurea coating material. After spraying a measured amount of the coating liquid, the final polyurea-coated urea (PCU) products were obtained. The polyurea synthesized by LU and PAPI coated urea is denoted as PN. The polyurea that was synthesized by LU, PAPI and polyether amine coated urea is denoted as PNA. PNE (PCU including ethylene glycol), PNG (PCU including diethylene glycol) and PNX (PCU including octanol) were obtained in the same way.

### N release behavior of PCUs

The N release behavior of PCUs in water at 25 °C was measured in accordance with the Chinese National Standard GB/T 23348-2009 (2009). For each sample, 10 g PCUs were sealed in a glass bottle containing 200 ml deionized water, and then incubated at 25 °C in incubator. After each incubated period (1, 3, 5, 7, 10, 14, 28, 42, 56, 84, 112 days), a certain amount of solution was taken out to measure the nitrogen content, then 200 ml deionized water was again added into the glass bottle and keep on incubating at 25 °C in incubator. The nitrogen concentration was measured using the Kjeldahl method (Bradstreet 1954). All samples were carried out in triplicate and the average value was taken as the nitrogen concentration of each sample. The N release longevity of PCUs is defined as the time when the cumulative N release reaches 80 % of the total N (Yang et al. 2013). The buried bag method was employed to determine the N release rate of PCU in soil (Hyatt et al. 2010). The buried experiments were accomplished according to the method of Yang et al. (2013).

### Morphology of PNG

Analyses were performed with a JSM-5800 scanning electron microscope (SEM). They were split into two halves, and the cross-section or surface of PNG was adhered to sample holders with double-sided adhesive tape. Prior to observation, the samples were coated with gold in a sputtering device.

### FTIR of coating materials

The PNG coating was analyzed using a Nicolet 380 FTIR spectrometer. The wavenumber range was from 500 to 4000 cm^−1^. Prior to analyses, Samples and KBr powder were ground and mixed uniformly for the preparation of tablets to obtain the FTIR spectra.

## Results and discussion

### Effect of different modifiers on N release characteristics

**Fig. 1:**
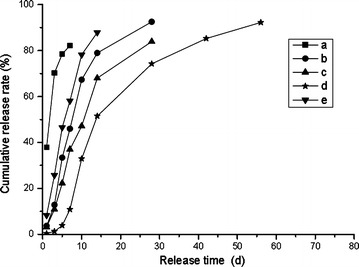
Cumulative nitrogen release curves of PCUs including the different modifier (*a* PN; *b* PNA; *c* PNE; *d* PNG; *e* PNX)

The granulated urea was firstly changed into a liquid urea (LU) to react sufficiently with PAPI. However, due to the urea molecular chain with low activity, the polyurea coating synthesized by LU and PAPI exhibited low crosslinking density and poor mechanical property. To improve the coating property, the different modifiers were added. Figure 1 shows the N release characteristics of PCUs with the different modifiers in coating. As shown in Fig. 1a, the controlled release ability of PN was poor, and the N release rate within 24 h was as many as 37.9 % and its N release longevity was only 6 days. It was related with the poor property of PN coating. To obtain the excellent property of coating, the amine compound polyether amine with the same groups as urea used as modifier was added into the coating to increase the chemical reaction between LU and PAPI. The controlled release ability of PNA (Fig. 1b) was improved obviously with the N release rate of 3.6 % within 24 h, but the N release longevity was still short (about 15.1 days), which did not meet the standard of controlled release fertilizers of the Chinese National Standard (30 days). Therefore, a series of alcohol compounds such as ethylene glycol, diethylene glycol, and octanol used as modifiers were added. The N release characteristics of PCUs (PNE, PNG, and PNX) were shown in Fig. 1c–e, respectively. The molecular weight and molecular chain of three alcohol compounds are gradually increased. However, the N release ability of the corresponding PCUs did not increase gradually, and their N release rate within 24 h was 3.1, 0.3, 8.2 % and the N release longevity was 24.4, 35.2, 10.8 days, respectively. It indicated that diethylene glycol was the best modifier for polyurea resin, and PNG exhibit excellent controlled release ability. The low molecular weight and molecular chain of alcohol compounds have no role on improving the property of polyurea coating. The higher molecular weight and longer molecular chain of alcohol compounds could hinder the chemical reactions between LU and PAPI, resulting in the increase of the water absorbency of coating. So, diethylene glycol is the best. Moreover, when other compounds were used as modifiers in the preparation of polyurea coating, PCU were not prepared successfully because the materials did not cure immediately in a few minutes. The coating technology is very particular and the thickness of coating is only 20–30 μm. It became tacky unless it cured for a few minutes, and if the uncured polyurea coating on the surface of PCU became tacky, all small urea particles would bond a big particle in a rotary drum. So, it is a high demanding to the coating material of PCU. Only alcohol compounds were used as modifiers in the preparation of polyurea coating (Zhang et al. 2014). Therefore, based on the above experimental results, diethylene glycol was used as the modifier of polyurea in the next discussion.

### FTIR analysis of coating materials

**Fig. 2:**
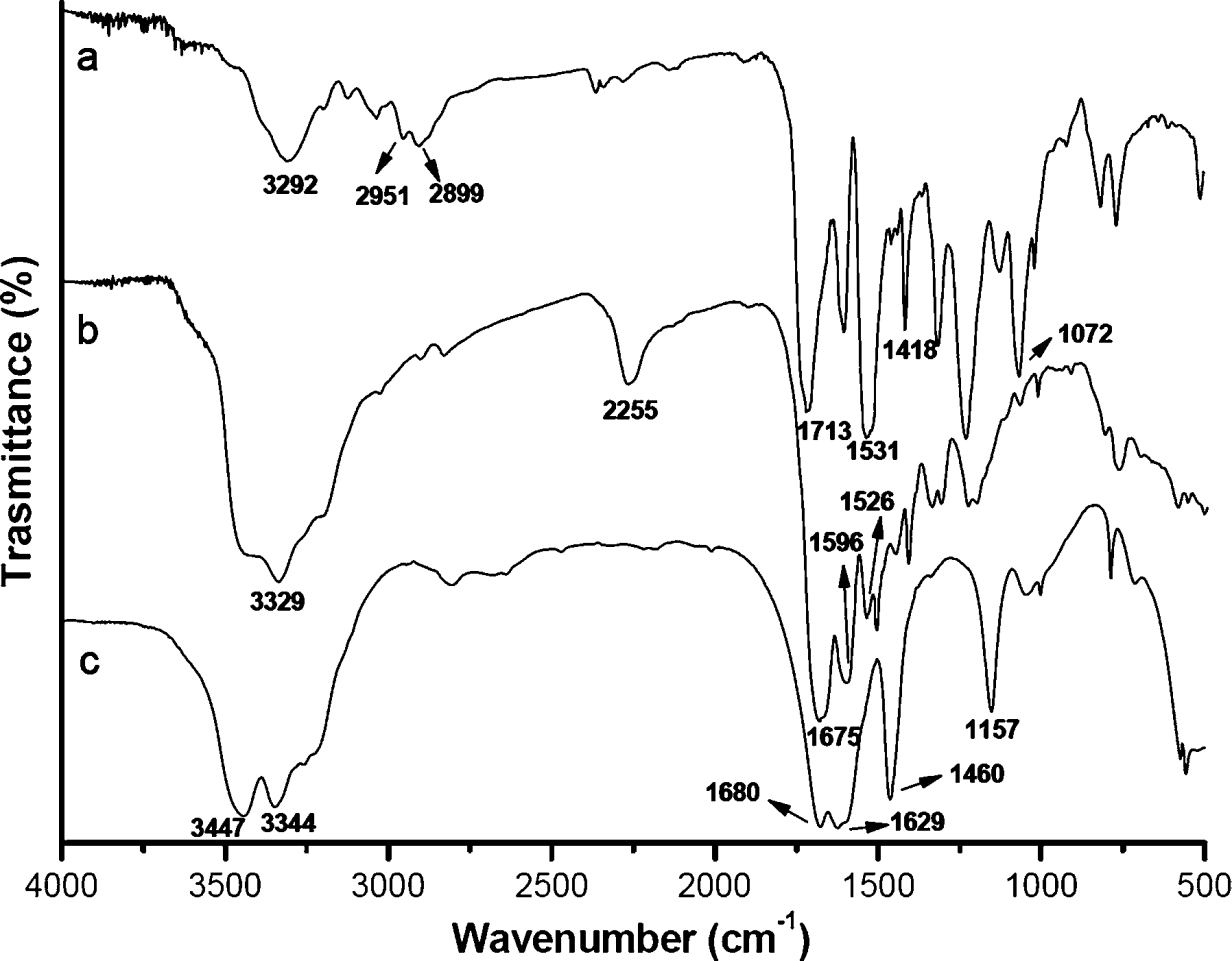
FTIR spectra of PNG (*a*), PN (*b*), and urea (*c*)

 Figure 2 shows the FTIR spectra of urea, PN, and PNG. In the FTIR spectra of urea (Fig. 2c), the presence of absorption peaks at 3344, 3447 and 1157 cm^−1^ can be attributed to the stretching vibration of N–H bonds. The peaks in the region of 1629–1680 cm^−1^ can be attributed to the C=O stretching bond of urea. The absorption peak for urea observed at 1460 cm^−1^ represents the stretching vibration of the C–N bond (Fig. 2c). The absorption peak for PN observed at 3329 cm^−1^ represents the stretching vibration of the N–H bond, and the peak at about 1675 cm^−1^ is due to the stretching vibration of the C=O bond (Fig. 2b). The 2255 cm^−1^ peak reflects the stretching vibration from the –N=C=O bond, and the absorption peaks at around 1596 and 1526 cm^−1^ might be associated with the *δ*N–H bonding. The results demonstrated the occurrence of the chemical reactions between the N–H of LU and NCO groups of PAPI and the formation of polyurea. The absorption peaks for PNG observed at 3292, 1531, 1713 cm^−1^ correspond to the stretching vibration of N–H, *δ*N–H and C=O bonds, respectively (Fig. 2a). In addition to these peaks, comparison with the FTIR spectra of PN, the FTIR spectra of PNG shows that new absorption peaks appear at 2951–2899, 1418, and 1072 cm^−1^. They can be assigned to νC–H, δC–H, and νC–O–C of diethylene glycol, respectively, indicating the existence of diethylene glycol in PNG (Fig. 2a).

### Morphology of PNG

**Fig. 3:**
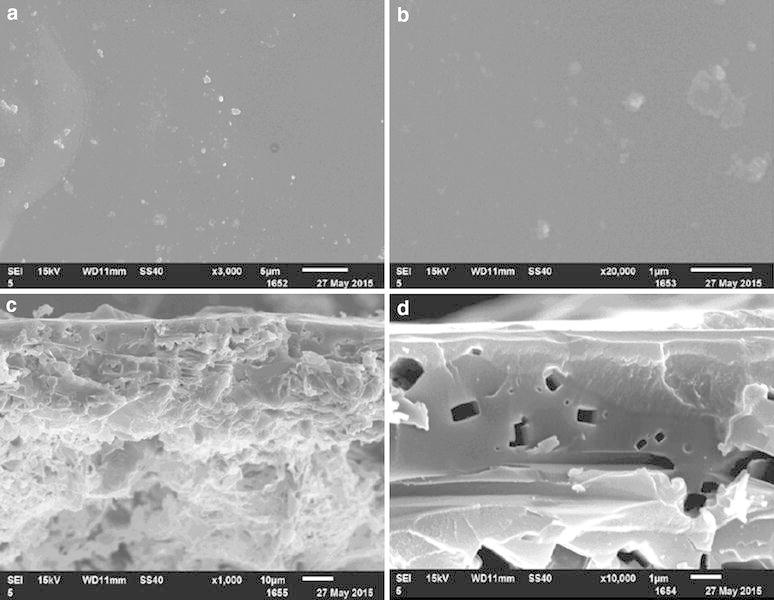
SEM images of PCU: **a** surface of PCU at ×3000 magnification; **b** surface of PCU at ×20,000 magnification; **c** cross section of PCU at ×1000 magnification; **d** cross section of coating materials at ×10,000 magnification

The SEM images illustrate the morphologies of PNG with the surface and its cross section in Fig. 3a–d. The surface of PNG coating shell (Fig. 3a) displays a compact and wrinkled appearance with some white urea powder caused inadvertently during the preparation of sample. When the surface were enlarged to 20,000 times (Fig. 3b), the coarse and compact surface were clearly observed. The SEM images of the cross section of PNG clearly showed the two-part structure with a clearly thin coating layer and the core urea (Fig. 3c), and the numerous gaps or pin holes were observed on the cross section of coating. When the SEM images were enlarged to 10,000 times, the size of these pin holes were in the range of 50–700 nm in Fig. 3d, and the pin holes can permit the free circulation of the solution between the interior and exterior of the shell. It indicated that the polyurea formed by the reaction of urea and PAPI was an excellent coating material for CRF with the modification of diethylene glycol.

### N release characteristics of PNG

**Fig. 4:**
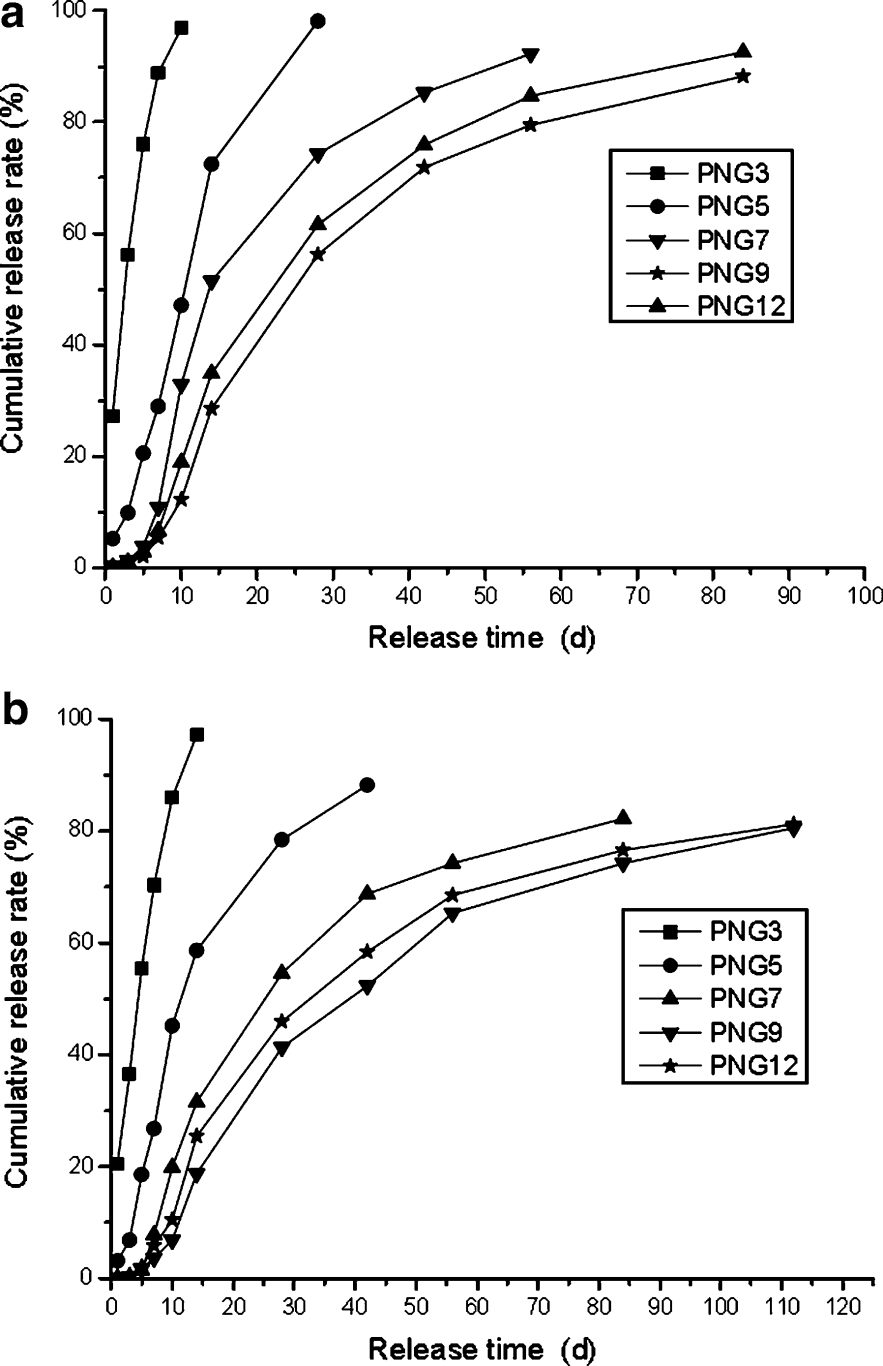
Cumulative nitrogen release curves of different coated fertilizers of PNG (the number beside PNG mean the coating rate in the coating materials of PNG) in water at 25 °C (**a**) and in soil (**b**)

The N release characteristics of PNGs in water at 25 °C were significantly affected by the different coating rates (Fig. 4a). The N release rates within 24 h for PNG3, PNG5, PNG7, and PNG9 were 27.25, 5.1, 0.32, and 0.01 % respectively. The N release longevity of the PNG3, PNG5, PNG7, and PNG9 increased gradually from 5.5 to 58 days with the coating rates increased from 3 to 9 %, respectively, indicating the gradual decrease of the cumulative N release rate of the PNGs (from PNG3, PNG5, PNG7 to PNG9) (Fig. 4a). However, the N release characteristic of PNG12 was similar to that of PNG9. It indicated that the controlled release ability of PNGs did not improve when the coating rate exceed 9 %, which was different from other studies (Yang et al. 2012). It was related with the property of the coating material. It was considered that the easily swollen coating material with the higher content after absorbing water had little or no effect on controlled release ability of CRF when the coating rate achieved a measured data. The polyurea belong to the type of coating material and the best coating rate for PNG is 9 %. It was also observed in Fig. 4a that the cumulative N release curves for PNGs changed from an “inverted-L” shape (PNG3 and PNG5, and PNG7) to an “S” shape (PNG9 and PNG12) with increasing the coating rate. The N release characteristics of PNGs in soil were shown in Fig. 4b, which is similar to that in water, indicating that N release rate of PNG in soil may be predicted according to that in water at 25 °C (Fig. 4a, b). Yang et al. (2012) also reported the similar results for CRF in water and soil.

### Biodegradability of coating materials

**Fig. 5:**
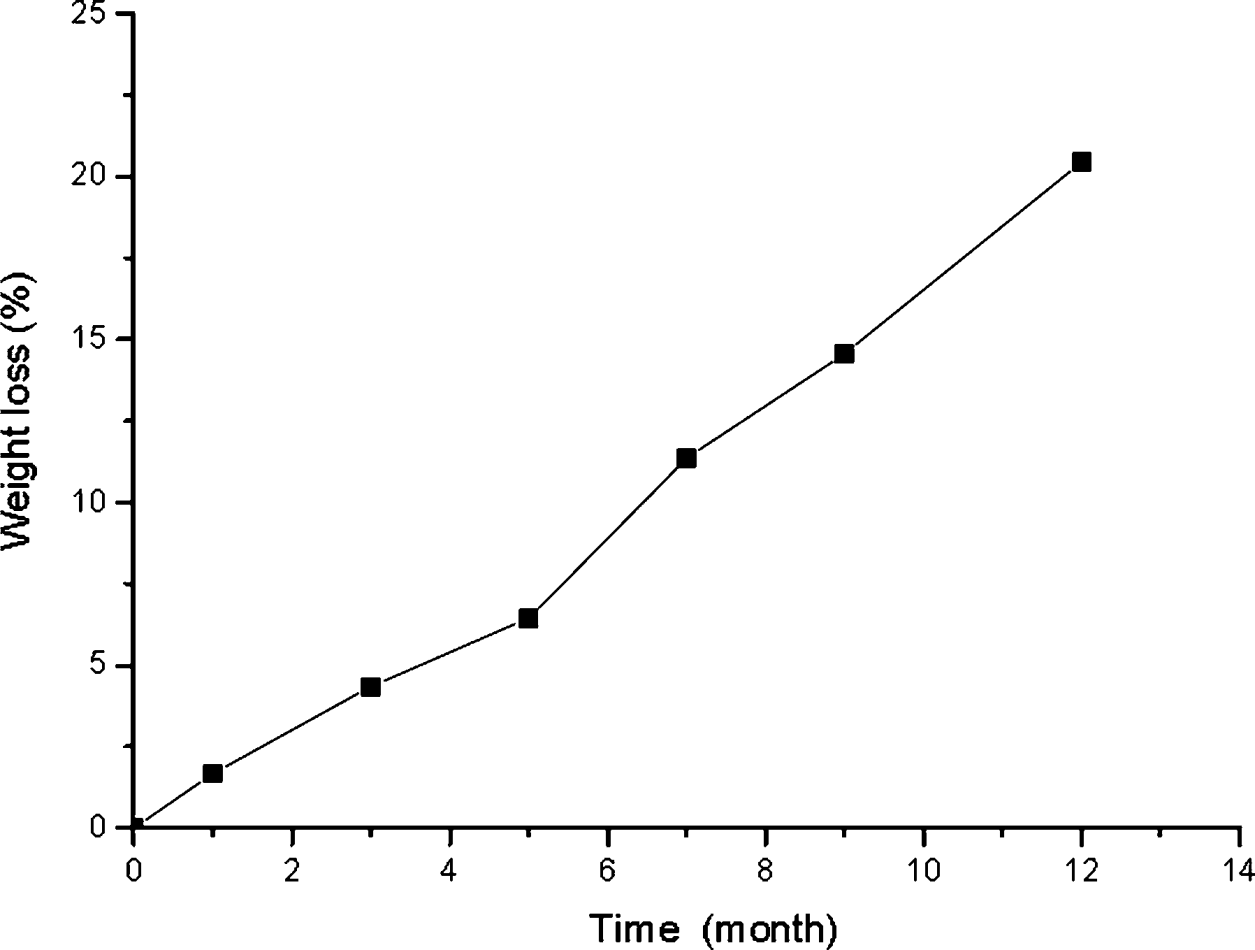
The weight loss of PCU after 12 months in soil

In this research, PNG coating were synthesized mainly from isocyanates and a liquid urea. After being buried in soil for 12 months, the residual coating debris diminished in size. We found that the ultimate weight loss exceeded 20 % (Fig. 5). The results clearly demonstrated the PNG coating material had an obvious trend of degradation in soil.

## Conclusions

A series of PCUs were prepared by coating urea with the polyurea synthesized by the reaction of PAPI with a liquid urea to reduce nitrogen release of coated fertilizers. The dense appearance and polyurea synthesis process for PCU coating were observed by SEM and FTIR. The PCU coating had excellent controlled-release ability, and the nitrogen release behavior was clearly improved with the addition of diethylene glycol. The PCU coating material had obvious trend of degradation in soil. These results show that the polyurea resin is an effective coating material of CRF.
